# Human Milk Oligosaccharides: Their Effects on the Host and Their Potential as Therapeutic Agents

**DOI:** 10.3389/fimmu.2021.680911

**Published:** 2021-05-24

**Authors:** Anaïs Rousseaux, Carole Brosseau, Sophie Le Gall, Hugues Piloquet, Sébastien Barbarot, Marie Bodinier

**Affiliations:** ^1^ INRAE, Biopolyméres Interactions Assemblages, Nantes, France; ^2^ INRAE, Bioressources: Imagerie, Biochimie & Structure, Nantes, France; ^3^ Centre Hospitalier Universitaire Nantes, UMR1280 PhAN, Nantes, France

**Keywords:** human milk oligosaccharides (HMO), microbiota, epithelial barrier, immune system, allergy

## Abstract

Breastmilk is known to be very important for infants because it provides nutrients and immunological compounds. Among these compounds, human milk oligosaccharides (HMOs) represent the third most important component of breastmilk after lipids and lactose. Several experiments demonstrated the beneficial effects of these components on the microbiota, the immune system and epithelial barriers, which are three major biological systems. Indeed, HMOs induce bacterial colonization in the intestinal tract, which is beneficial for health. The gut bacteria can act directly and indirectly on the immune system by stimulating innate immunity and controlling inflammatory reactions and by inducing an adaptive immune response and a tolerogenic environment. In parallel, HMOs directly strengthen the intestinal epithelial barrier, protecting the host against pathogens. Here, we review the molecular mechanisms of HMOs in these different compartments and highlight their potential use as new therapeutic agents, especially in allergy prevention.

## Introduction

**Figure 1:**
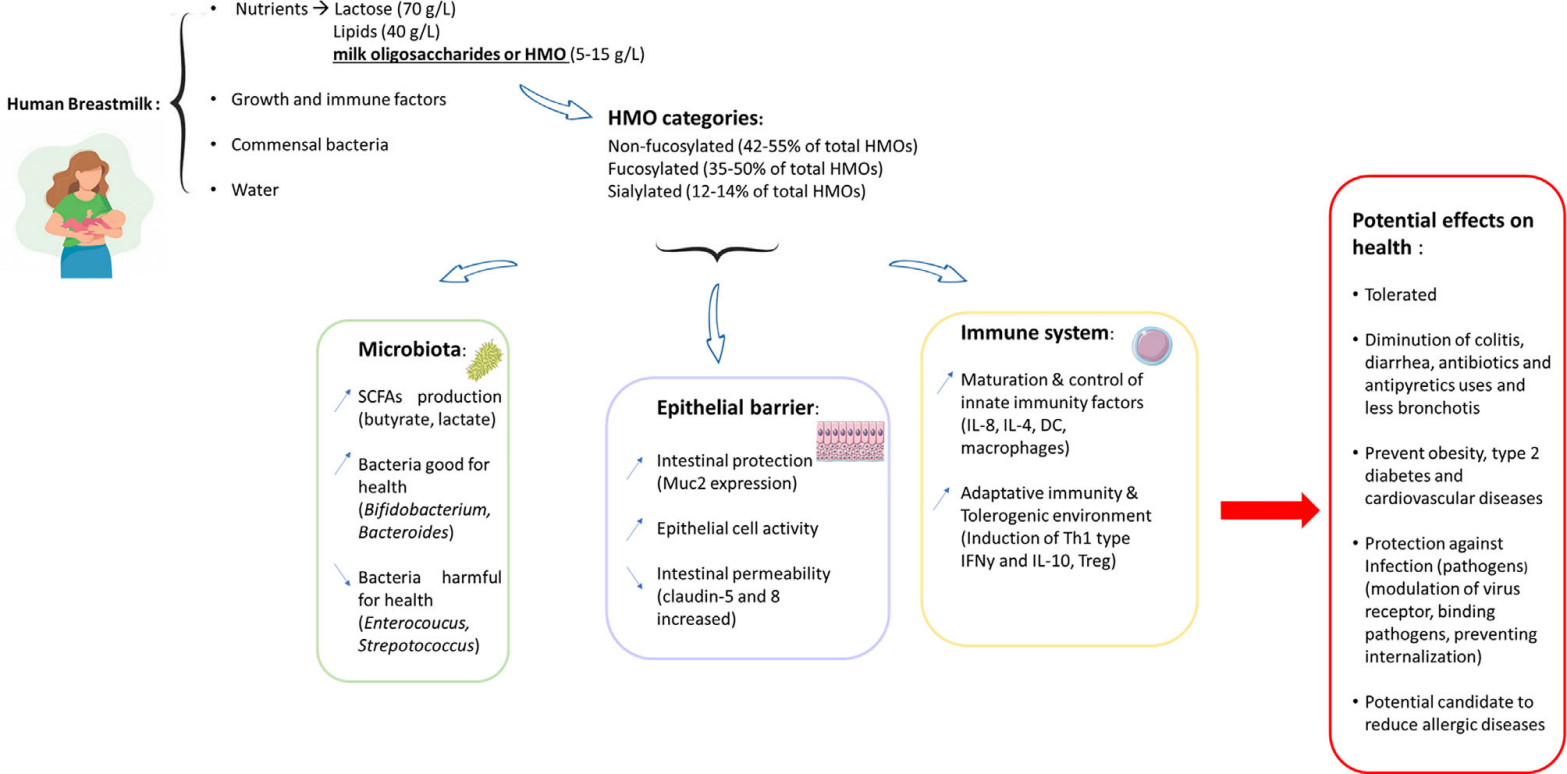
Graphical abstract: beneficial effects of HMOs on human health.

Breastmilk plays a crucial role in the development of children and provides initial protection against pathogens (1). Breastmilk is composed of nutritional and immunological compounds such as proteins, lipids, and carbohydrates as well as cytokines, growth factors, antimicrobial compounds (1) and microbes (2). All these compounds are transferred directly from the mother to the infant during breastfeeding. According to the World Health Organization (WHO), breastmilk is the ideal food and provides everything essential for the proper development of the child (1). Indeed, some components of breastmilk are able to modulate the biological systems of the child, especially the microbiota, epithelial barriers and immune system. These modulations have a real impact on the child’s health and allow the establishment of functional biological systems essential for adulthood. One of the main components naturally found in breastmilk is oligosaccharides, called human milk oligosaccharides (HMOs). The discovery of HMOs is the result of cooperation between two disciplines: medicine and biology. It started at the end of the 19th century when researchers observed that breastfed infants survived better and had fewer intestinal diseases than formula-fed infants (3) (**Figure 1**).

## Structure of HMOs and Their Compartmentalization in Mothers and Children

**Figure 2:**
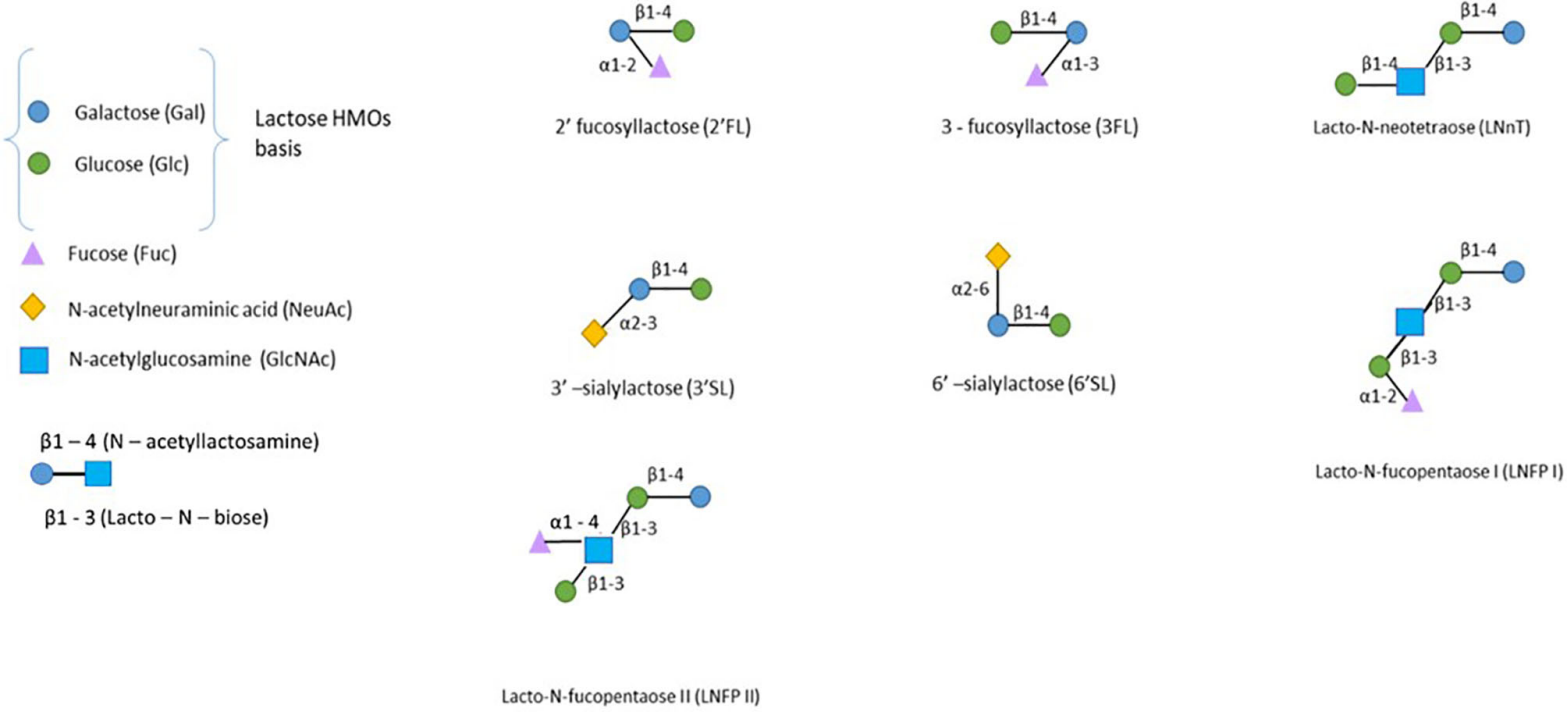
The structure of human milk oligosaccharides. HMOs can be classified into neutral fucosylated HMOs (2’-FL, 3FL, and LNFPI-II), neutral HMOs (LNnT) and sialylated HMOs (3’SL and 6’SL). Only the most prevalent HMOs are presented here (4).

HMOs are complex oligosaccharides derived from the elongation of a lactose backbone (Galβ(1–4)Glc) at the nonreducing end galactose (Gal), at the reducing end glucose (Glc) by a fucose residue (linked in α1-2/α1-3) and/or at the nonreducing end Gal by a sialic acid residue (linked in α2-3/α2-6) or by disaccharides lacto-N-biose I (linked in β1-3) or *N*-acetyllactosamine (linked in β1-3/6) (**Figure 2**) (5). Additional elongation can occur, and more than 150 HMOs have been described to date (5).

HMOs in breast milk are usually classified regarding their -osidic composition as fucosylated neutral HMOs (35-50% of total HMOs), nonfucosylated neutral HMOs (42-55% of total HMOs) and sialylated HMOs (12-14% of total HMOs) (6).

HMO concentrations in milk are dependent on several factors. Their concentration varies during lactation from 20-25 g/L in colostrum to 5-20 g/L in mature milk (7). Indeed, 2’-fucosyllactose (2’-FL) and lacto-N-fucopentaose (LNFP) are especially present during early lactation, whereas 3’-fucosyllactose (3’-FL) increases across the lactation period (8). Conversely, the lacto-N-neotetraose (LNnT) concentration in milk decreases during lactation, including 2’-FL and LNnT (9). Sialylated HMOs are more abundant in colostrum (approximately 1018 mg/kg of fresh milk or milk from baby food companies), are less concentrated in transitional and mature milk (approximately 696 and 365 mg/kg of fresh milk or from baby food companies, respectively) and are not found in late lactation milk (10).

The composition of HMOs in human milk is also dependent on genetic factors, including secretor and Lewis blood status of women (**Table 1**) (21, 22). The secretor gene (*Se*) encodes an α1-2-fucosyltransferase (FUT2), and the Lewis gene (*Le*) encodes an α1-3/4-fucosyltransferase (FUT3), allowing the fucosylation of HMOs. Fucosyltransferase polymorphisms generated by the individual capacity of women to express these enzymes lead to four genetic expression profiles: Se+Le+ (Lewis a-b+), Se-Le+ (Lewis a+b-), Se+Le- (Lewis a-b-) and Se-Le- (Lewis a-b-). For example, women without functional FUT2 cannot produce 2’-FL or LNFPI (21). This lack of HMOs may induce consequences for metabolic activities, especially diarrheal diseases, in infants (23). On the other hand, women without functional FUT3 cannot produce HMOs with α1,3/4 fucosyl linkages such as 3’-FL and LNFPII (24). Recently, glucose levels in the blood of the mother were shown to affect the composition of HMOs in breastmilk. Indeed, the presence of sialylated HMOs is associated with a low concentration of fasting plasma glucose and insulin in nonsecretor mothers. In secretor mothers, insulin sensitivity is associated with a high concentration of difucosyllactose and LNFPII (25).

**Table 1:** Overview of studies used in this review demonstrating the role of HMOs in host protection (clinical trials).

Study	Workforce	Intervention	Efficiency
Morrow et al. (11) Observational clinical trial	93 breast-feeding motherinfant pairs from birth to 2 years	Diarrhea was diaenosed by a study physician. Milk samples obtained 1to 5 weeks postpartum were analyzed for oligosaccharide content. Data were analyzed by Poisson regression.	*Campylobacter* diarrhea occurred less often in infants whose mother's milk contained high levels of 2'-FL. Colicivirus diarrhea occurred less often in infants whose mother's milk contained high levels of lacto-N-difucohexaose (LDFH-I).
Marriage et al. (12) Randomized and prospective clinical trial	Infants were exclusively fed either formula (n = 189) or Human Milk (n = 65)	In healthy, singleton infants (birth weight ≥2490 g). Formula-fed infants were randomized to 1 of 3 formulas with a caloric density of 64.3 kcal/dl. Each formula contained galactooligosaccharides, and the 2 experimental formulas contained varying levels (0.2 and 1.0g/L)of the HMO 2'-fucosyllactose (2'FL).The 3 formula groups were compared with an HM-fed reference group	Growth and 2'-FL uptake were similar to those of HM-fed infants
Kajzer et al. (13) Prospectvie, randomized, multi-center, double-blinded, controlled 3-arm tolerance study in full term, singleton infants (birth weight ≥ 2490g) enrolled between 0 and 8 days of age	Experimental Formula 1 (EF1) did not contain oligosaccharides (n=42) and Experimental Formula 2 (EF2) contained 2g/LscFOS and 0.2./L2'FL(n=46). The 2 formula groups were compared with a human milk-fed (HM) reference group (n=43)	Infants were exclusively fed formula or human milk from enrolment untli 35 days of age Data related to intake, stool patterns, anthropometrics and parental questionnaires were collected. The primary outcome was average mean rank stool consistency (MRSC) from Study Day 1 to Visit 3. MRSC was calculated from stool records (1=watery, 2=loose/mushy,3=:soft, 4=formed, 5=hard).	2'-FL and fructo-oligosaccharides fed from less than eight days of age for approximately one month was well tolerated; stool consistency, anthropometric data, and frequency of feedings with spitting up/vomiting and was similar to that of infants given formula without oligosaccharides or to infants breastfed
Sprenger et al. (9) An observatory, single center, longitudinal cohort study	50 mothers, who gave birth to 25 female and 25 male singleton infants	Quantitative human milk collection at 30, 60, and 120 days postpartum from 50 mothers, who gave birth to 25 female and 25 male sineleton infants. Quantification of the 5 representative HMOs: 2'FL, Lacto-N-tetraose (LNT), Lacto-N-neotetraose (LNT), 3'Sialyllactose (3'SL) and 6'Sialyllactose (6'SL). We grouped the milk samples and corresponding infants according to the measured milk 2'FL concentrations at 30 days of lactation, which clustered around low concentrations (95% Cl of mean 12-42 mg/L) and high concentrations (95% Cl of mean 1880-2460 mg/L) with the former likely representing Secretor negative mothers. Infant anthropometric measures were recorded at birth, 1, 2 and 4 months of age. Relations among the quantified HMOs and the relation of the high and low 2'FL HMOs groups with infant growth parameters were investigated via linear mixed models	2'-FL doesn't influence height, weight, BMI, and head circumference of the infants who consumed breast milk with low or high FUT2 associated HMO concentrations and composition
Puccio et al. (14) A Randomized Multicenter Trial	Healthy infants, 0 to 14 days old, were randomized to an intact-protein, cow's milk-based infant formula (control, n = 87) or the same formula with 1.0g/L2'fucosyllactose (2'FL) and 0.5 g/L lacto-N-neotetraose (LNnT) (test,n = 88) from enrollment to 6 months	All infants received standard follow-up formual without HMOs from 6 to 12 months. Primary endpoint was weight gain through 4 months. Secondary endpoints Included additional anthropometric measures, gastrointestinal tolerance, behavioral patterns. and morbidity through age 12 months	2'-FL and LNnT were well-tolerated and supported age·appropriate growth. Gastrointestinal symptoms (flatulence, spitting up. and vomiting) were similar between the groups. Enfants receiving formula with 2'-FL and LNnT had significantly softer stools, and infants born by caesarian section also had a lower incidence of colic at four months of age. Infants fed the formula with 2'-FL and LNnT compared to Infants fed the formula without HMOs had significantly fewer parental reports of bronchitis, reduced incidence of lower respiratory tract infections, reduced use of antipyretics and reduced use of antibiotics
Kuhn et al. (15) Early weaning trial in 958 HIV-infected women	Nested case-cohort analysis of mortality to 2 y of age among 103 HIV-infected and 143 HEU(uninfected) children	Breast-milk samples collected at 1mo postpartum were analyzed for HMO content. Samples were selected to include mothers of al HIV·infected children detected by 6 wk of age, of whom 63 died at <2 y of age; mothers of all HEU children who died at <2 y of age (n = 66); and a random sample of 77 HEU survivors. Associations before and after weaning in HIV-infected and HEU infants separately were investigated by using Cox models	2'-FL reduce mortality in HIV non infected infant from HIV infected mother
Seppo et al, (16) A prospective birth cohort designed to assess immunologic factors in human milk, development of CMA within the first18 months of life and oversampled for newborns at high risk for food allergies	Non-CMA infant n = 41 CMA infant = 39	The earilest available milk sample was assessed from each mother; at median 1.0 months in 41 mothers of non-CMA (cow's milk allergy) infants and at median 1.4 in 39 mothers of CMA infants. CMA was verified by oral food challenges at median 6 months of age. HMO composition was measured by HPLC after 2-aminobenzamide labeling. Raffinose was added to milk samples as internal standard to allow for absolute quantification.	Enfant fed with breatsmilk with high level of LNPIII are protected against cow's milk allergy compared tp children fed with breatsmilk containing low level of LNPIII.
Sprenger et al. (17) Randomized controlled trial	FUT2-dependent oligosaccharides in breast milk samples of mothers (n = 266) from the placebo group of a randomized placebo-controlled trial of prebiotics and probiotic as preventive against allereic disease in infants with high allergy risk (trial registry number: NCT00298337).	Using logistic regression models, we studied associations between FUT2-dependent breast milk oligosaccharides and incidence of allergic disease at 2 and 5 years of age.	Infants born by C-section and having a high hereditary risk for allergies might have a lower risk to manifest lgE-associated eczema at 2 years, but not at 5 years of age, when fed breast milk with FUT2 -dependent milk oligosaccharides.
Miliku et al., (18) CHLID cohort	421 mother-infant dyads from the Canadian Healthy Infant Longitudinal Development (CHILD) cohort	Associations of 19 individual HMOs and overall HMO profiles with food sensitization at 1 year of age using Projection on Latent Structures-Discriminant Analysis (PLS-DA)	HMOs composition in the human milk is associated with food sensitization in the first year of life could lead to a food allergy later
Nowak-Wegrzyn et al., (19) Randomized controlled trial	Of the 82 children with CMPA that were screened, 67 (intention-to-treat [ITT] cohort-mean age 24.5 ± 13.6 months; range 2-57; 45 [67.2%] male ) were randomized to receive either the Test or the Control formula during the first DBPCFC	A whey-based EHF (Test formula) containing 2'fucosyl-lactose (2'FL) and lacto-N-neotetraose (LNnT) was assessed for clinical hypoallergenicity and safety. The Control formula was a currently marketed EHF without HMO. Children with CMPA (cow's milk protein allergy), aged 2 months to 4 years, were assessed by double-blind, placebo-controlled food challenges (DBPCFC) to both formulas, in randomized order. If both DBPCFC were negative, subjects participated in a one-week, open food challenge (OFC) with the Test formula. Symptoms and adverse events were recorded. Hypoallergenicity was accepted if at least 90% (with 95% confidence intervals) of subjects tolerated the Test formula.	Hydrolyzed formula supplemented with 2'-FL and LNnT met the clinical hypoalleragenicity criteria and can be recommended for the management of cow's milk protein allergy in infants and young children
Lodge et al., (20) the Melbourne Atopy Cohort Study	Colostrum and early lactation milk samples were collected from 285 mothers enrolled in a high-allergy-risk birth cohort, the Melbourne Atopy Cohort Study	Nineteen HMOs were measured. Profiles/patterns of maternal HMOs were determined using LCA. Details of allergic disease outcomes including sensitization, wheeze, asthma, and eczema were collected at multiple follow-ups up to age 18 years. Adjusted logistic regression analyses and generalized estimating equations were used to determine the relationship between HMO profiles and allergy	Compared with children exposed to the neutral Lewis HMO profile, exposure to acidic Lewis HMOs was associated with a higher risk of allergic disease and asthma over childhood, whereas exposure to the acidic-predominant profile was associated with a reduced risk of food sensitization

Furthermore, HMOs composition in breastmilk is also dependent on the geographical locations in the world. Gomez-Gallego et al., studied the milk composition from 79 healthy women from Finland, Spain, South Africa, and China (26). They showed that in Chinese sample LNFPIII and 3’-FL are more abundant compared to Finland sample but LNFPI and 2’-FL are more abundant in Finland and Spain samples compared to Chinese and South Africa samples. A higher abundance of 3’-FL is also observed in South Africa sample compared to Finland and Spain samples.

The vast majority of HMOs reach undigested the large intestine where they provide selective substrates for specific gut bacteria, modulate the immune system, and prevent the epithelial adhesion of intestinal pathogens (27). A small proportion is absorbed intact and excreted in the urine. The presence of intact HMO in the urine of breast-fed infants was first described in 1996 by Rudloff and colleagues (28), with subsequent clinical studies reporting absorption rates of 1–5% HMO from the gut intestinal tract to the circulatory system (29). Weather HMOs are absorbed in the upper gastrointestinal tract and/or in the large intestine is unknown. Because it was demonstrated that the small intestine is the main site of nutrient absorption (30), we can postulate that HMOs absorption occurs mainly in the upper gastrointestinal tract but further investigation *in vitro* need to be performed with representative cell lines of the colon or the small intestine. Experiments using Ussing Chamber Assay, performed on different part of the small intestine or colon to estimate the uptake of the HMOs could be considered.

Interestingly, HMOs concentrations in the child urine or in the circulatory system correlate with the levels in the mother’s milk. HMO contribute then to the development and physiological functions of other organs and systemic cell systems. Indeed, while fractions of absorbed HMOs are low, these concentrations were shown to have biological effects *in vitro* and could explain some of the benefits of human milk (31).

In conclusion, it was shown that HMOs are present in maternal serum during pregnancy (32). HMO concentration and composition in the serum varies according to secretor status and gestational age but also according to geographic or environmental factors (33). 2′-FL seems to be produced between the end of the first trimester and mid-pregnancy, contributing to an increase in total HMOs in Se+ women. Another study demonstrated that HMOs are also present in cord blood and suggested the placental transfers of HMOs from the maternal bloodstream to the fetal bloodstream (34).

## HMOs Effects on the Gut Epithelial Barrier

The intestinal barrier is constantly renewing itself through the production of epithelial cells at the base of the villi (35). These cells migrate into the intestinal lumen and differentiate throughout their ascent along the villi. Some of these cells will allow the absorption of nutrients (enterocytes), and others will produce mucus (goblet cells) to protect epithelial cells against pathogens present in the lumen (35). The proliferation of epithelial cells must be controlled because overactivation of cell proliferation could induce intestinal cancer (36).

### HMOs Effects on Epithelial Cells

**Figure 3:**
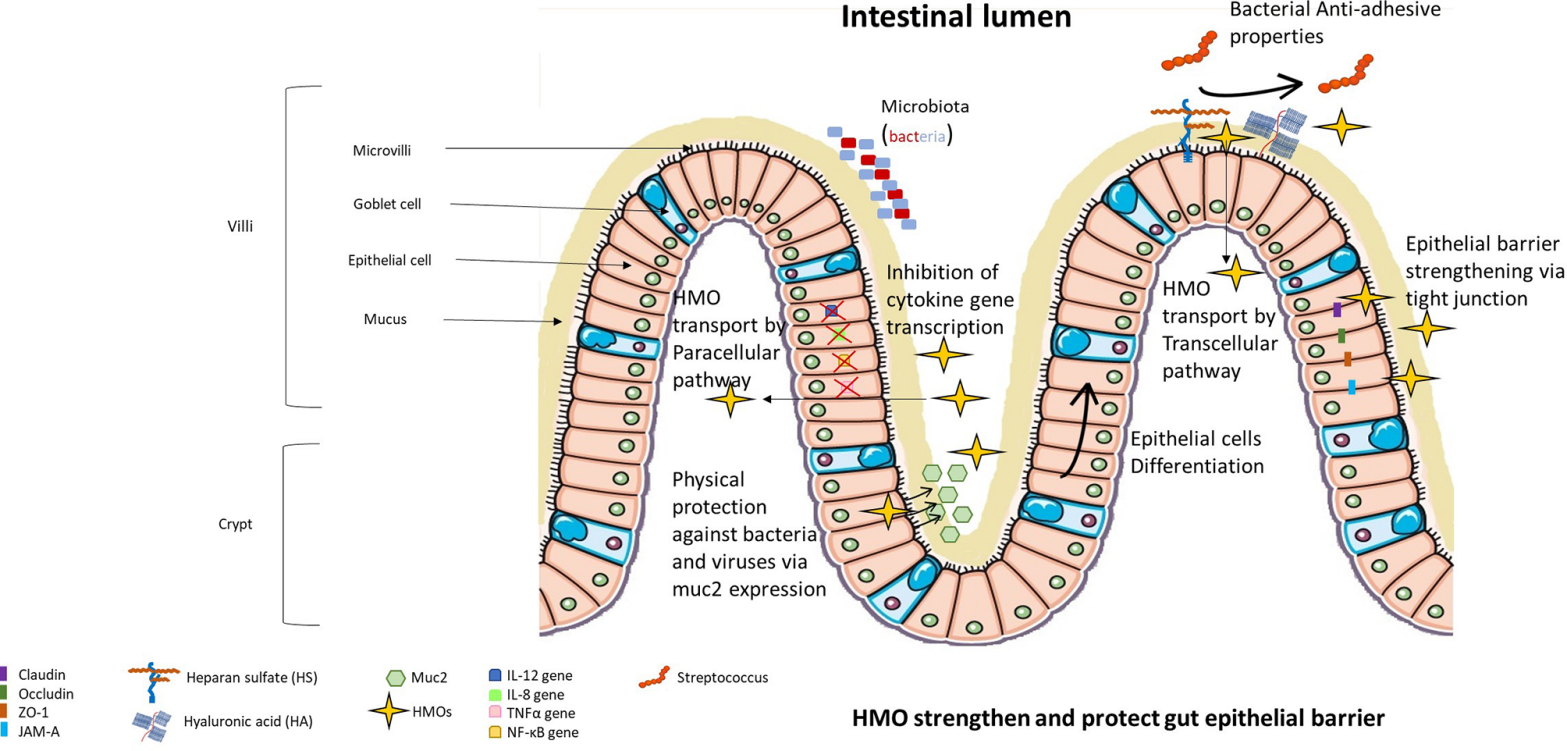
HMOs strengthen and protect the gut epithelial barrier. HMOs are able to modify the extracellular glycocalyx of epithelial cells, limiting the adhesion of bacteria and viruses to epithelial cells. HMOs can protect the intestine due to their capacity to amplify the secretion of muc2 (main protein constituting the mucus protecting epithelial cells) and their capacity to strengthen tight junctions. A small portion of HMOs can be absorbed and thus can modulate gene expression inside epithelial cells, limiting their proliferation and inducing their differentiation into villus enterocyte-type cells. Absorbed HMOs also limit the expression of proinflammatory cytokines.

First, Angeloni et al. demonstrated that sialylated HMO fractions (especially 3’SL) induce a modification of the extracellular glycosylation pattern of the Caco-2 cell line (37). This modification was associated with a 50% reduction in enteropathogenic *E. coli* in Caco-2 cells treated with 3’-SL compared to untreated cells (**Figure 3**). Moreover, Chunli Kong et al. demonstrated that 3’-FL increased albumin absorption and the presence of heparan sulfate (HS) and hyaluronic acid (HA) in the glycocalyx of a Caco-2 cell line (38).

In 2008, Kuntz et al. observed that neutral and acidic fractions of HMOs induced cell growth inhibition of small intestinal epithelial cells (HIECs), HT-29 cells and Caco-2 cells *in vitro* (39). Furthermore, these two fractions of HMOs induced HT-29 and HIEC differentiation but not Caco-2 cell differentiation. Finally, only neutral HMOs induced HT-29 and HIEC apoptosis characterized by caspase 3 cleavage but not Caco-2 cells supporting the hypothesis of differentiation-associated processes. They concluded that HMO are able to induce growth inhibition in intestinal cells through two different mechanisms, by suppressing cell cycle progression through induction of differentiation and/or by influencing apoptosis. By inducing the differentiation of intestinal cells *in vitro*, HMOs may influence the maturation of the intestinal barrier (**Figure 3**). Indeed, Holscher et al. determined that neutral HMOs induced the differentiation of HT-29 cells in villus enterocyte-like cells, promoting digestive capacities of the epithelial barrier (40). Finally, Perdijk and colleagues confirmed that sialylated HMOs influence intestinal cell homeostasis *in vitro*, highlighting the beneficial effect of HMOs on epithelial cells (41).

In conclusion, some studies demonstrated that HMOs are able to modify the extracellular glycosylation of epithelial cells limiting the adhesion of some pathogens and enhancing albumin absorption. A couple of another studies agree on the effects of HMO on the modulation of the cell cycle preventing cell growth and inducing cell differentiation in villus enterocyte-like cells leading to a potential better absorption of nutrients. As the development and maturation of digestive and absorptive processes depend on differentiation, studies show that HMOs are effective at influencing various stages in gastrointestinal development.

### HMOs Effects on the Function of Intestinal Barrier

HMOs can also protect the intestine by inducing the production of mucin (**Figure 3**). Mucin is the main protein that composes intestinal mucus and protects against intestinal infection (42). Indeed, Wu et al. demonstrated that exposure of human intestinal epithelial cells and intestinal organoids to HMOs induced expression of Muc2, a chaperone protein, that then activated disulfide isomerase (PDI) *in vitro* (43). They also showed an increase in Muc2 expression and a decrease in intestinal permeability in an HMO-supplemented murine model.

Suligoj et al. demonstrated in their study that 2’-FL alone or 2’-FL mixed with LNnT induced the expression of the claudin-8 gene in Caco-2 cell monolayers and that the fermentation of 2’-FL induced the expression of claudin-5 in gut-on-chips from human organoids derived from proximal, transverse, and distal colon biopsies (44). Claudin proteins play a crucial role in tight junctions between two epithelial cells, blocking the passage of molecules in the paracellular pathway and thus decreasing the permeability of the gut (45) (**Figure 3**).HMOs incubated with *B. longum infantis* increased mRNA transcription of ZO-1 and the junctional adhesion molecule (JAM-A) in Caco-2 cells and HT-29 cells (46). Furthermore, the authors demonstrated that HMOs prevent the internalization of occludins inside Caco-2 and HT-29 cell lines (46). However, Sua Kim et al. demonstrated that ZO-1 and occludins are necessary to maintain transepithelial electrical resistance and thus to preserve the function of the proximal tubular epithelium (47). ZO-1 (and ZO-2) are involved in the regulation of tight junction assembly, and JAM-A proteins regulate the structural organization of tight junctions, which is essential for the impermeability of the epithelial barrier (48).

In conclusion, these studies highlight the efficiency of HMOs to protect and strengthen the impermeability of intestinal barrier by modulating mRNA transcription and protein expression of several key molecules such as ZO-1, muc2 and claudin.

### HMOs Effects on the Activity of Epithelial Cells

HMOs can affect the protein expression of epithelial cells because a small portion of HMOs are absorbed by intestinal cells (**Figure 3**). Acidic oligosaccharides were shown to only pass through intestinal cells *via* the nonspecific paracellular route, and neutral oligosaccharides were shown to pass through intestinal cells *via* the paracellular or transcellular route involving the receptor pathway (49). The action of HMOs on gene expression was also demonstrated *in vivo*, showing that supplementation with sialylated HMOs in rats affects intestinal gene expression and modulates the intestinal glycome (50).Sialylated HMOs reduce the secretion of IL-12 in Caco-2 cells and decrease the gene expression of IL-12, IL-8, and TNFα (51). NF-κB gene expression and protein translocation were also reduced by HMOs in that study. These effects are mediated by the induction of the nuclear receptor PPARγ, leading to the regulation of anti-inflammatory peptidoglycan recognition protein 3 activation. It was also shown in HT-29 and Hep-2 cell lines that 2’-FL induced a decrease of 80% in *C. jejuni* invasion, and this decrease was associated with suppression of mucosal proinflammatory cytokine secretion (IL-8, IL-1β and neutrophil chemoattractant macrophage inflammatory protein 2) (52).

One *in vitro* study revealed that 2’-FL could have promising beneficial effects on allergy signaling pathways by selectively inhibiting IL-8 and CCL20 release by T-84 and HT-29 in response to the antigen-antibody complex in the PPARγ-dependent pathway (53) (**Figure 3**).

In conclusion, all these studies converge to demonstrate that HMOs reduce pro-inflammatory cytokines secretion by epithelial cells.

## HMOs Promote the Proliferation of Specific Intestinal Bacterial Strains and the Secretion of Short-Chain Fatty Acids

The infant microbiota is essentially transmitted from the mother at parturition and during lactation (54). Microbial ecosystems are essential for the good health of the child and need to be developed quickly after birth and during the first months of life (55). The first body site to be colonized is the intestinal tract (GI) (55) by aerobic bacteria (for example *Enterococcus*). Oxygen consumption by these bacteria then promotes the colonization of anaerobic bacteria in the colon (for example *Bacteroides, Clostridia*, and *Bifidobacteria*). Nevertheless, the microbial system of children is extremely variable in terms of composition and stability before becoming more stable in adulthood (56). HMOs influence the composition of the intestinal tract microbiota by selecting for some bacterial species (56), such as *Bifidobacterium* and *Bacteroides*, which indirectly prevents the colonization of other bacterial strains in the infant gut (55). Some *in vitro* and *in vivo* studies were conducted to evaluate the effect of HMOs on gut bacteria.

### *In Vitro* Studies

Zhuo-Teng Yu et al. revealed that 2’-FL and 3’-FL induced *Bifidobacteria* and *Bacteroides* growth *in vitro*. Conversely, these 2 HMOs do not generate major growth effects in *Lactobacillus, Enterococcus*, and *Streptococcus* (57). Additionally, 3’SL and 6’SL induced moderate growth of *Bifidobacteria* and *Bacteroides* (57), two commensal bacteria known to be good for human health (58–60). Conversely, *Enterococcus* (61) and *Streptococcus* (62) are two bacteria that induce human infection. Other *in vitro studies* were conducted and demonstrated that HMOs are able to decrease the colonization (63) and growth (64) of group B *Streptococcus*. Moreover, Salli and colleagues demonstrated that 2’-FL induces an increase in members of the phyla Firmicutes and Actinobacteria *in vitro* (65); *Bifidobacteria* that are commonly found in the gut belong to the phylum Actinobacteria (66). In this study, 2’-FL induced a decrease in members of the Proteobacteria phylum that known to cause different diseases (67). Finally, Hoeflinger et al. demonstrated that no Enterobacteriaceae strains grew in the presence of 2’-FL, 6’SL or LNnT (68). In conclusion, all *in vitro* studies agree that HMOs not only induce the emergence and proliferation of bacteria that benefit health but also decrease the frequency of harmful bacteria.

Interestingly, a very recent study demonstrated that HMOs such as 2’-FL and LNT are hydrolyzed by Akkermansia muciniphila (69). This bacterium is known to colonize the mucus layer of the human intestinal tract and is associated with decreases in obesity, type 2 diabetes, inflammatory bowel disease, and appendicitis (69).

To summarize, these *in vitro* studies highlight the ability of HMOs to modulate the microbiota inducing on the one hand the growth of bacteria strains benefic for the health and on the other hand the decrease of bacteria strains involved in bacterial infections.

### *In Vivo* Studies

**Figure 4:**
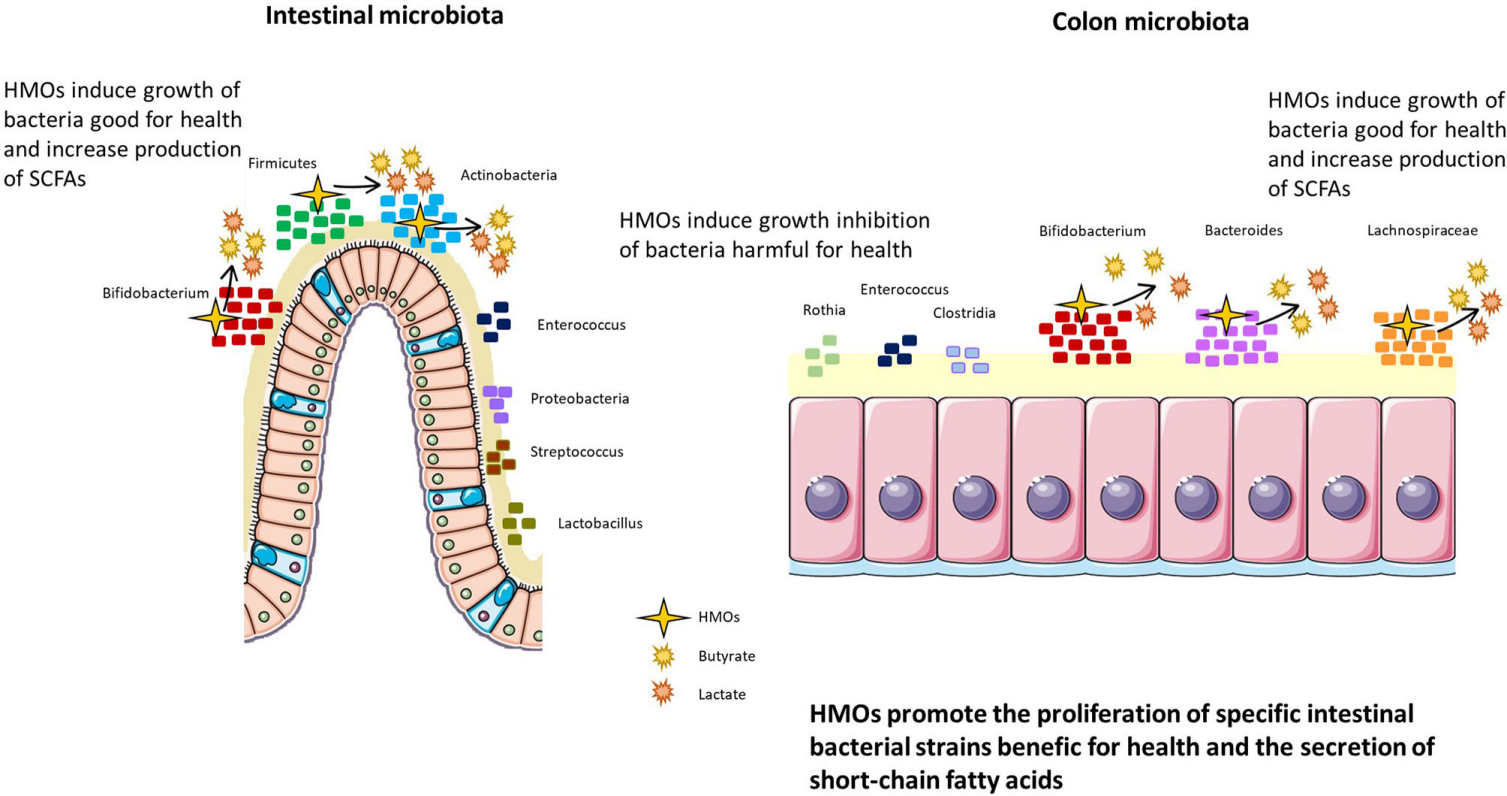
HMOs promote the proliferation of specific intestinal bacterial strains beneficial for health and promote the secretion of short-chain fatty acids. HMOs modulate the intestinal and colon microbiota, inducing the proliferation of Bifidobacterium, Firmicutes, and Actinobacteria in the intestine and Bifidobacterium, Bacteroides, and Lachnospiraceae in the colon. In parallel, HMOs decrease populations of bacteria harmful to health, such as Enterococcus, Proteobacteria, Streptococcus, and Lactobacillus in the intestine and Rothia, Enterococcus, and Clostridia in the colon.

Two *in vivo* studies were conducted in newborn pigs supplemented with 2’-FL (10 g/L for 7 days), and both revealed a trend towards an increase in the *Lachnospiraceae* genus and a decrease in *Enterobacteria* in the intestine, inducing a nonsignificant diminution of diarrhea in newborn pigs (70, 71) (**Figure 4**).

In clinical trials in humans, HMOs indicated their ability to modulate the microbiota of breastfed preterm infants and breastfed caesarian-born infants, altering the microbiota to be more similar to that of breastfed to term vaginally born infants (increasing Firmicutes and *Bifidobacteria* and decreasing *Enterococcus*) (72, 73). Interestingly, *Bifidobacteria* and *Bacteroides* were found in the infant fecal microbiota of one-month-old breastfed children only if their mother secreted 2’-FL and LNFP in milk (74). This finding was correlated with limited infection (75) and protection against obesity (76). HMOs also have an influence on the colon microbiota and are directly fermented by bacteria from the colon (74) (**Figure 4**). Indeed, the composition of the microbiota is different depending on the HMOs consumed (74). For example, the consumption of all HMOs (nonfucosylated neutral, fucosylated, and sialylated) by one-month-old children induces a higher abundance of *Bifidobacteria* than the consumption of only one type of HMO (74). Furthermore, Wang et al. highlighted the correlation between HMO consumption and fecal microbiota composition (77). Indeed, 2’-FL supplementation was positively correlated with the abundance of *Bacteroides* and negatively correlated with *Veillonella*, *Enterococcus*, and *Rothia* in the feces of infants at 3 months postpartum.

As previously mentioned, the mother secreting status influences the HMO composition in breast milk. Therefore, the presence or absence of HMOs modulates the infant microbiota (78). It was shown that infants fed by secreting mothers had a higher relative abundance of *Bifidobacterium* strains and *Bacteroides* strains in feces than infants fed by nonsecretor mothers. Additionally, infants fed by secreting mothers presented fewer *Enterobacteria*, *Clostridia*, and *Streptococci* than infants fed by nonsecretor mothers. Another clinical trial demonstrated that *Prevotella* was not detected in stools from secretor mothers and their children (79). Similar to previous studies, *Bifidobacterium* abundance was increased in the stools of children from secretor mothers compared to children from nonsecretor mothers, and this effect was still relevant at 2 to 3 years of age.

Finally, it was shown that the consumption of acidic HMOs protects against microbial structure changes due to stress (80), and neutral HMOs are a selective substrate for some strains of *Bifidobacteria* (81).

In conclusion, clinical trials demonstrate the importance of the secretor status of mothers for the presence of HMOs in breastmilk and in the composition of the infant microbiota. These clinical trials correlate with the beneficial effects of HMO postnatal consumption and diverse and rich microbiota compositions that are beneficial for health.

### Secretion of Short-Chain Fatty Acids

HMOs can also modify the fermentation activity of the gut microbiota, especially short-chain fatty acid (SCFA) production (**Figure 4**). SCFAs are an important source of energy for enterocytes and are key signaling molecules for the maintenance of gut health. HMOs can indirectly increase the production of SCFAs, and these augmented levels are mediated by *Bifidobacteria* species such as *B. infantis* (82), *B. bifidum* (83) and *B. breve* (84). Other bacteria able to ferment HMOs such as Bacteroidetes, produce abundant lactate and other SCFAs and consumption of 3’SL and 6’SL by *Bifidobacterium* and *Bacteroides* induces greater neuraminidase activity (57). High neuraminidases activity was nevertheless, shown to be involved in human disorders such as neurodegenerative disorders, cancers, and infectious diseases (85). Furthermore, HMOs increase butyrate production in the colon thanks to their fermentation activities (44) (**Figure 4**).

Human trials and experimental murine models have wildly shown that increasing SCFAs through a high-fiber diet or direct supplementation have beneficial health effects. However, the effects of too high SCFAs concentration on the host has to be investigated, has one study found that higher SCFA concentrations were associated with obesity and hypertension in the adult (86).

## Effects of HMOs on the Immune System by Direct or Indirect Effects

In the period directly after birth, the neonate’s cellular immune system undergoes rapid development. Multiple immune factors, such as neutrophils, macrophages, monocytes, natural killer (NK) cells, T cells, and dendritic cells (DCs), play a crucial role in fighting against invading pathobionts (87). At birth, an imbalance between Th1/Th17 and Th2 phenotypes is noted in T helper (Th) cell populations. A neonate’s immune system is directed more towards the Th2 phenotype, which promotes humoral immunity and confers protection against extracellular pathogens. On the other hand, Th1/Th17 pathways target intracellular pathogens and are less prevalent in developing neonates (Adkins, 2000). The immature immune system is also characterized by overexpression of inflammatory markers and inadequate feedback regulation of immune signaling. It is hypothesized, therefore, that breast-milk oligosaccharides favorably modulate neonatal innate immune responses by controlling the expression of inflammatory markers involved in cell trafficking and by affecting cytokine and chemokine networks that regulate Th1/Th2 lymphocyte balance (88). However, information regarding the role of HMOs and the specificities of their action in inflammation are limited and are largely dependent on findings from *in vitro* and *ex vivo* studies.

### Direct and Indirect Mechanisms of HMOs on the Immune System

**Figure 5:**
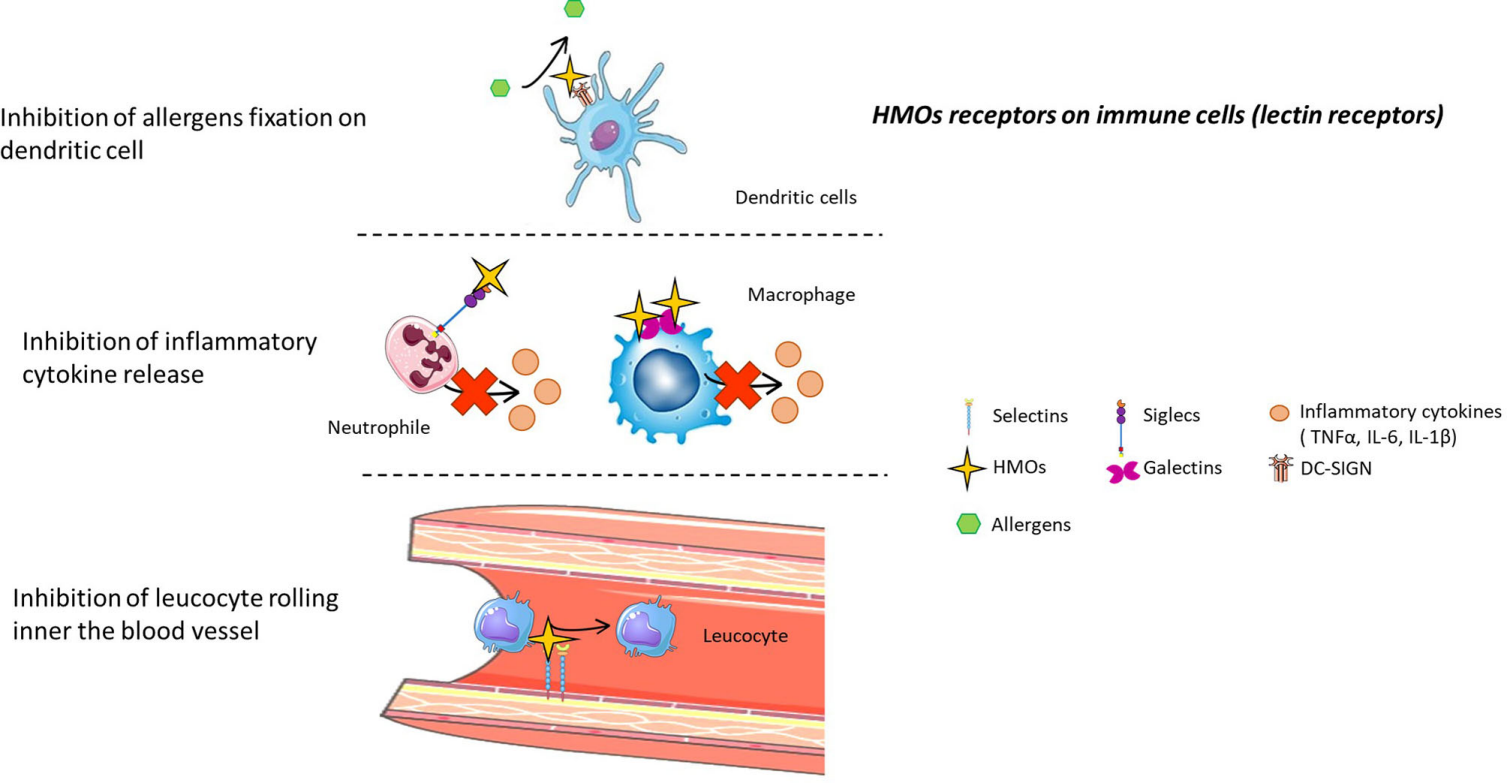
HMO receptors on immune cells (lectin receptors). HMOs can directly interact with the immune system by binding to several receptors present on immune cells. Thus, HMOs can prevent allergic asthma, limit inflammatory cytokine release, and inhibit the rolling of leucocytes inside blood vessels by competing with substrates for these receptors.

Because HMOs mediate changes in the composition of the infant microbiota or in the intestinal epithelial cell response, they may indirectly affect the infant immune system. Many *in vitro* studies suggest that HMOs can also directly modulate immune responses (**Figure 5**). Indeed, HMOs may act either locally, on cells of mucosa-associated lymphoid tissues, or at a systemic level, as 1-5% of HMOs are absorbed and reach the circulatory system (89).

#### Indirect Effects of HMOs on the Immune System *via* Microbiota and SCFAs

HMOs can indirectly modulate the immune system by acting on the microbiota, highlighting the important communication between these two biosystems.

HMOs induce the proliferation of *Bacteroides fragilis*, a bacterial strain that can induce the development of FOXP3^+^ regulatory T cells *via* a specific “inducible genetic signature” *in vitro* (90). This was also confirmed *in vivo*, in which colonization of the mouse intestine by this species increased the production of polysaccharide A, inducing the conversion of CD4^+^ effector T cells into FOXP3^+^ regulatory T cells producing the anti-inflammatory cytokine IL-10 (90). Furthermore, lactate produced after HMO fermentation allows the CD4^+^ T cells to switch to IL-17^+^ T cell subsets and reduces the cytolytic capacity of CD8^+^ T cells, modulating the inflammatory immune response (91). In addition, HMO fermentation also produces butyrate, which in turn participates in the regulation of innate and adaptative immune cell generation, trafficking and function (92). Butyrate also inhibits the recruitment and proinflammatory activity of neutrophils, macrophages, dendritic cells and effector T cells. Finally, butyrate is involved in the increase in regulatory T cell number and activity (92).

In conclusion, indirect effects of HMOs on the immune system tend to promote a tolerogenic environment characterized by an increased regulatory immune balance. These studies conducted *in vivo* are observational and highlight the correlation between HMOs supplementation and a higher rate and function of regulatory cells. However, the direct link and the proper molecular mechanism have to be deciphered *in vitro*.

#### Direct Effects of HMOs on the Immune System *via* Lectin Receptors

##### Galectin Receptors

HMOs can bind some galectin receptors, such as galectin-1, 2, 3, 7, 8, and 9 (93). These galectin receptors are expressed by immune cells such as dendritic cells, macrophages, granulocytes and mast cells (94) (**Figure 5**).

##### Siglec Receptors

Sialylated HMOs can bind sialic acid binding immunoglobulin-like lectins (Siglecs), notably Siglecs 5 and 9 (4). These receptors are found on neutrophils, monocytes and dendritic cells, and activation of these receptors after ligand binding leads to the apoptosis of neutrophils, limiting inflammation (95) (**Figure 5**).

##### Selectin Receptors

It was demonstrated that acidic fractions of HMOs in blood inhibit rolling and adhesion of leukocytes (monocyte, lymphocyte and neutrophil cells) to endothelial cells *in vitro* (96). Sialylated HMOs present epitopes of selectin ligands, sialyl Lewis (x) and sialyl Lewis (a), and thus bind selectin inside the blood vessel, preventing selectin-mediated emigration of leukocytes (97) (**Figure 5**). Sialylated HMOs can also reduce the formation of platelet-neutrophil complexes (98). Indeed, this complex is mediated by selectin interactions, and thanks to their epitope, sialylated HMOs prevent the interaction between neutrophils and platelets, limiting infectious disease (**Figure 5**). Lactodifucotetraose (LDFT) also inhibits platelet adhesion to collagen-coated surfaces, preventing their aggregation and activation and thus preventing the release of proinflammatory molecules (99). Finally, it was demonstrated that adjacent neutrophil activation, such as β2 integrin expression, was decreased when cells were treated with HMOs (98).

##### DC-SIGN

Fucosylated HMOs bind C-type lectin receptors named DC-SIGN receptors (100). DC-SIGN receptors, present on the surface of both macrophages and dendritic cells, are involved in house dust mite allergy because their activation by different major house dust mite allergens induces the secretion of proinflammatory cytokines (101). Thus, HMOs could prevent asthma, allergic rhinitis and atopic dermatitis by binding DC-SIGN and preventing its activation by allergens (**Figure 5**).

While direct binding of HMOs and receptors was highlighted in various studies *in vitro*, it would be interesting to inhibit the binding (by siRNA or knock-out for example), to fully determine the impact of the HMOs on the functions of the cells.

### HMOs Act on the Immune System, Stimulating Innate and Adaptive Responses

#### HMOs Activate a Controlled Inflammatory Innate Response

**Figure 6:**
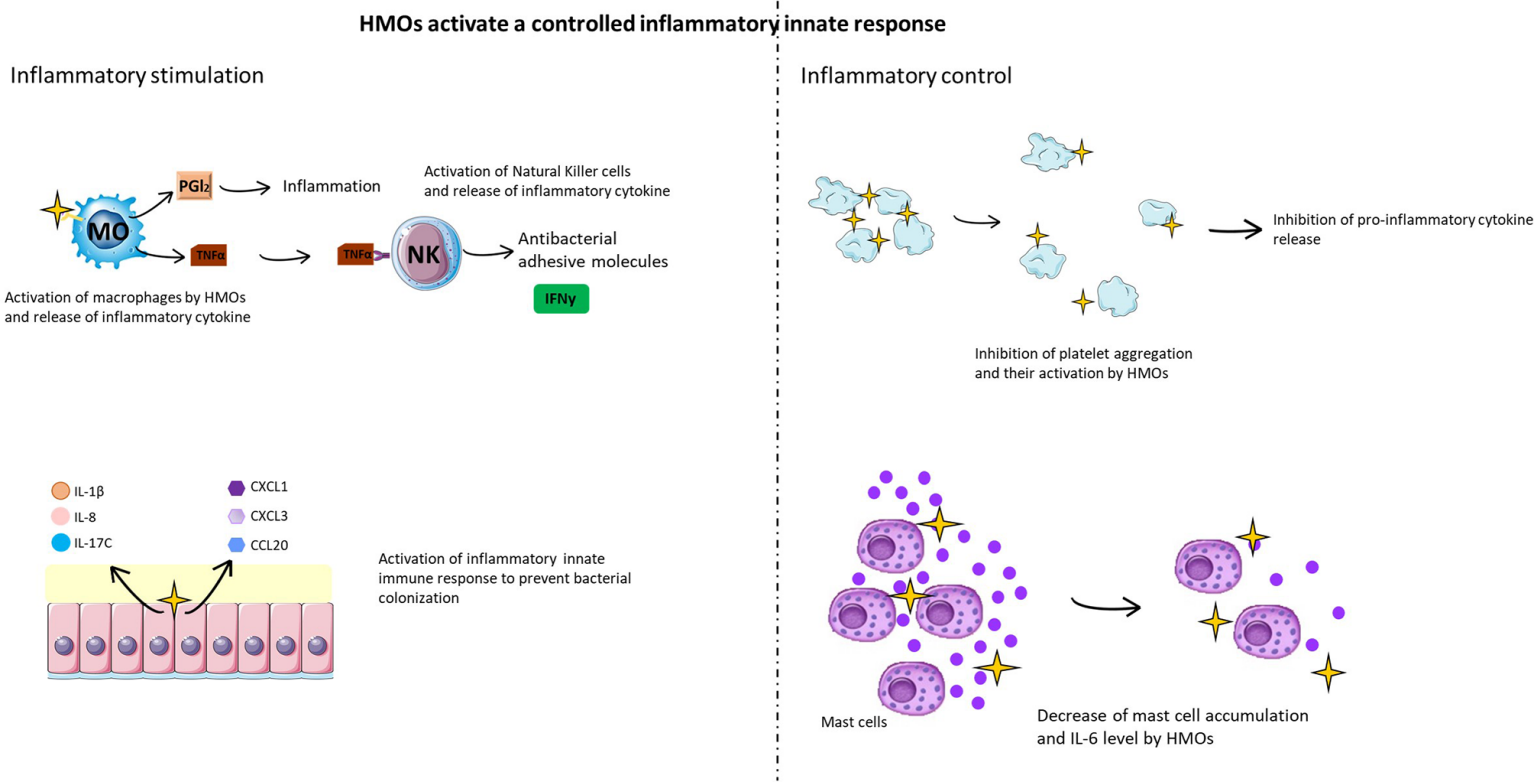
HMOs activate a controlled inflammatory innate immune response. HMOs participate in the inflammatory immune response, stimulating cellular (macrophages, mast cells, natural killer cells, and platelets) and molecular (cytokines and chemokines) actors of the innate response to fight against infection (left figure). In parallel, HMOs limit this inflammatory reaction in a cellular and molecular feedback loop (right figure).

Some HMOs are able to promote the inflammatory response by acting on proinflammatory cytokine secretion (**Figure 6**). Indeed, a study conducted by Lane et al. (102) demonstrated that HMOs are able to activate the expression of cytokines (IL-1β, IL-8, IL-17C and platelet factor 4), chemokines (CXCL1, CXCL3, CCL20, CXCL2, CXCL6, CCL5, CX3CL1 and CXCL2) and cell surface receptors (IFNγR1, ICAM-1/2, and IL10Rα) from an HT-29 cell line (colonic epithelial cells) (**Figure 6**). Usually, the activation of proinflammatory cytokines and chemokines, such as CXCL1, CXCL3, CCL20, IL-8, IL-1β, and IL-17C, traduces the establishment of the innate immune response to prevent bacterial colonization (102). LNFPIII and 3’SL have proinflammatory functions mediated by the TLR4 signaling pathway, leading to ERK/MAPK pathway activation (103). LNFPIII is able to mature dendritic cells into a type 2 phenotype, leading to the release of the proinflammatory cytokines IL-4 and IFNγ (104). LNFPIII can also activate macrophages *in vitro* (105), leading to the secretion of prostaglandin 2 and tumor necrosis factor alpha (TNF-α). These cytokines activate natural killer (NK) cells, inducing an increase in CD69 expression and interferon gamma (IFNγ) production directly at the inflammatory site.

Concerning the effects of HMOs on DC cells, the results are not similar between all studies, probably because of the nature of the HMOs used or the type of DC investigated. Isolated HMOs were found to stimulate semimaturation of human monocyte-derived DCs, which was associated with elevated levels of anti-inflammatory cytokines such as IL-6, IL-10, and IL-20 (106). Ayechu-Marabuzu and colleagues showed that monocyte-derived DCs exposed to 2’-FL induced IFNγ and IL-10 secretion by CD4+ T cells and also induced a Th1-driven immune response (107). Another study demonstrated that 3’SL directly stimulates mesenteric lymph node CD11c+ DCs, inducing the production of cytokines implicated in Th1 and TH17 responses (108). HMOs were also found to reduce LPS-induced production of proinflammatory cytokines such as IL-12p70 and TNF-α by preventing the interaction of LPS and TLR4 (106). Zhang et al. provided further mechanistic insight by demonstrating that a neutral HMO fraction influenced inflammatory cell populations *via* nuclear factor (NF)-κB and mitogen-activated protein-kinase pathways. Finally, another study performed by Perdijk et al. revealed that HMOs have no effects on the differentiation or maturation of DCs *in vitro* (109).

However, it is important to mention that this activation of the inflammatory response is controlled. For example, an *in vitro* study demonstrated that lactodifucotetraose (LDFT) inhibits platelet adhesion to collagen-coated surfaces, preventing their aggregation and activation and thus inhibiting the release of proinflammatory molecules (99) (**Figure 6**). It was also demonstrated that adjacent neutrophil activation, such as β2 integrin expression, was decreased when cells were treated with HMOs (98). Additionally, 2’-FL and 3’-FL have anti-inflammatory effects mediated by the TLR4(CD14)/STAT3/SOCS2 and TLR3 signaling pathways, respectively (105). DSLNT also has an anti-inflammatory effect, but the effect of TLR has not yet been determined. Finally, in a rat model of necrotizing enterocolitis infection, supplementation with the sialylated HMO fraction induced a decrease in mast cell accumulation (**Figure 6**), DPPi activity and IL-6 levels in ileal tissue compared to non-supplemented rats (110). It was also shown that mice supplemented with 2’-FL presented a high decrease in the *C. jejuni* infection rate (80%), a reduction in weight loss, and a decrease in intestinal inflammation and inflammatory signal induction (52).

Several studies have focused on assessing the immunomodulatory effects of HMO mixtures isolated from human milk. For example, He et al. assessed the impact of colostral HMOs on intact immature human intestinal mucosa (111). The authors found that colostral HMOs reduced the levels of pro-inflammatory cytokines (such as interleukin (IL) IL-1β, IL-6 and IL-8) while stimulating the expression of cytokines associated with tissue repair and homeostasis.

In conclusion, it seems clear that the effects of HMOs on immune cells depend on the nature of the HMOs. Therefore, elucidating the effects of each HMOs is require to fully understand the mechanistic effects leading to a controlled inflammatory innate response. While the effects of HMOs were well investigated concerning DCs cells, further studies are require on the direct effects of HMOs on other major cellular actors of the innate response such as NK cells or innate lymphoid cells. Finally, *in vitro* experiments are require to fully understand the molecular mechanism of HMOs on these cells and to make a link between molecular pathways induced and inhibition of inflammatory innate response.

#### HMOs Activate the Adaptive Immune Response

**Figure 7:**
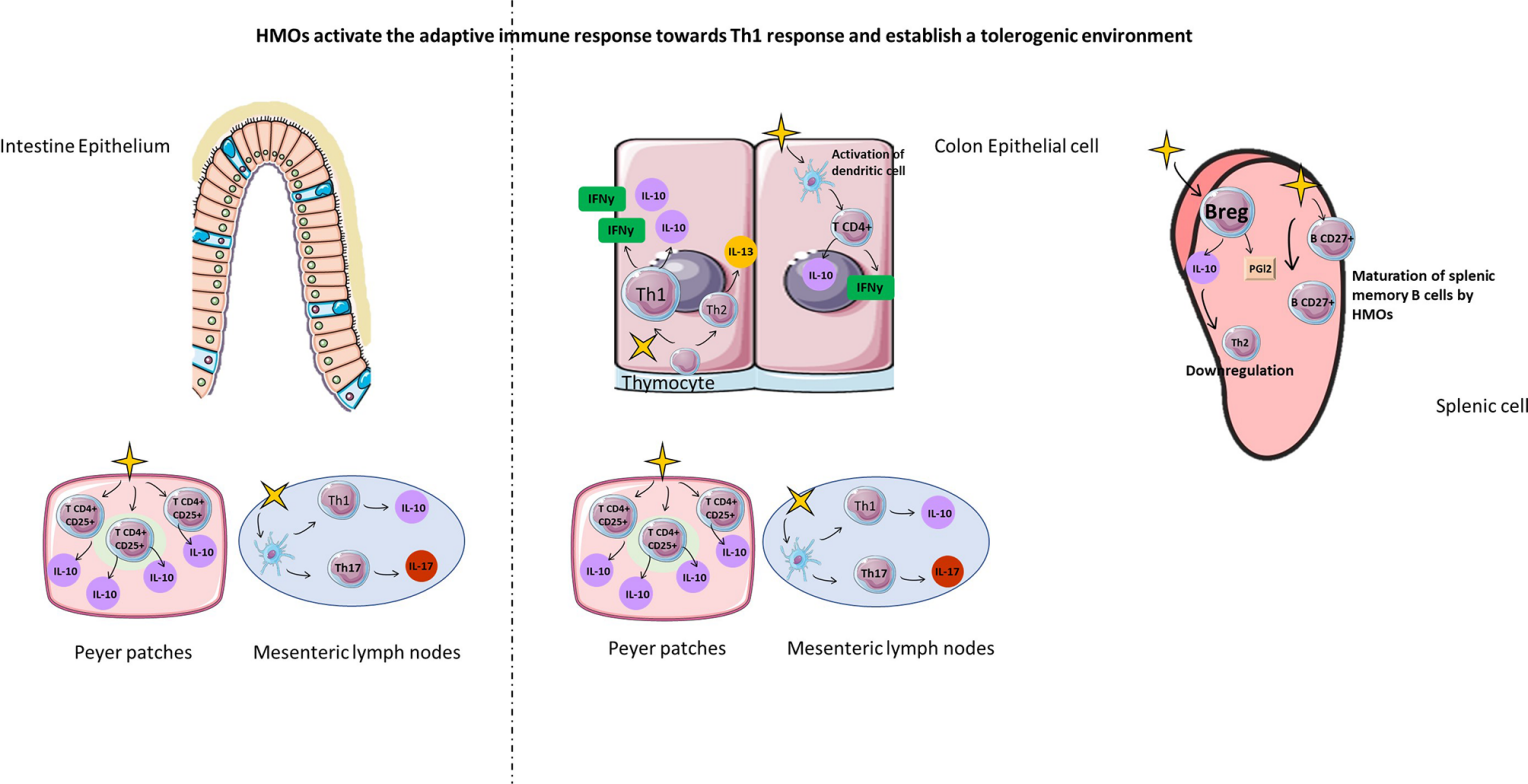
HMOs activate the adaptive immune response towards the Th1 response and establish a tolerogenic environment. HMOs can also act on cellular actors in the adaptative immune (Th1, TH17, TCD4+, Treg, Breg, memory B cells) response in order to fight against infectious diseases with a downregulation of Th2 immune response in the intestine and in the spleen. Furthermore, HMOs are able to introduce a tolerogenic environment with stimulation of Treg and Breg cells.

HMOs are able to induce a tolerogenic immune response acting on immune cells and molecular factors. Recently, Ayechu-Muruzabal et al. demonstrated *in vitro* that 2’-FL was able to induce Th1 type IFNγ and regulatory IL-10 secretion of peripheral blood mononuclear cells, whereas Th2 type IL-13 was reduced compared to cells in the absence of 2’-FL (107). He et al. also found that exposure to HMOs elevated the expression of Th1 polarization and shifted the balance of Th1/Th2 cytokines towards that of more mature tissues (111). In addition, 2’-FL activated immature monocyte-derived dendrites, leading to the secretion of IFNγ and IL-10 by CD4^+^ T cells (107) (**Figure 7**). Another *in vitro* study investigating the gastrointestinal epithelial transfer of HMOs showed that HMOs can first transfer through a Caco-2 cell monolayer and then significantly suppress Th-2-type cytokine production by Ara h1-specific CD4^+^ T cells from patients allergic to peanuts (112). Interestingly, supplementation with LNFPIII in a mouse model of schistosome infection induced the proliferation of splenic non-T cells and B220+, CD4-, and CD8- cells secreting IL-10 and prostaglandin 2 (113) (**Figure 7**). This proliferation leads to a downregulation of the Th2-type immune response, limiting an exacerbated inflammatory reaction. Finally, 3’SL also induces the production of cytokines secreted by Th1 and Th17 cells by direct stimulation of mesenteric lymph node CD11c^+^ dendritic cells (108) (**Figure 7**).

Furthermore, the regulation of inflammation by 2’-FL was also demonstrated *in vivo* and was characterized by an increase in IL-10^+^CD4^+^CD25^+^ regulatory T cells from Peyer’s patches (**Figure 7**). Xia et al. demonstrated *in vivo* that mice supplemented with 2’-FL had increased expression and activity of the CD27 marker on splenic B cells compared with control mice, highlighting the maturation of B cells (114) (**Figure 7**). They also observed the proliferation of proinflammatory CD4+ CD8+ splenic T cells with a higher concentration of IFNγ in supplemented mice than in control mice (114) (**Figure 7**). Activation of inflammation by HMOs may appear at an early stage of neonate development, as demonstrated by Eiwegger and colleagues. Interestingly, they showed that acidic HMOs increased the expression of IFNγ by CD3^+^CD4^+^ and CD3^+^CD8^+^ cord blood T cells and IL-13 expression by CD3^+^CD8^+^ cord blood T cells (115). Furthermore, Eiwegger et al. demonstrated that the acidic fraction of HMOs increased CD25 expression on CD3^+^CD4^+^ cells characteristic of activated regulatory T cells (115).

One study conducted by Sotgiu et al. revealed that the presence of 2’-FL and LNFPI decreased the LPS-activated mononuclear cell proliferation obtained from multiple sclerosis and healthy patients (116). These 2 HMOs reduced the production of IL-12 and IFNγ more significantly in multiple sclerosis patients than in healthy patients. Conversely, IL-10 was increased in both patients. An important clinical trial conducted by Goehring et al. demonstrated that breastfed and formula-fed children with 2’-FL had a similar low concentration of proinflammatory cytokines (IL-1rα, IL-1α, IL-1β, IL-6, and TNFα) compared to children that were not breastfed or supplemented with HMOs at 4 months of age (117).

In conclusion, HMOs are able to induce a tolerogenic immune response acting on immune cells and molecular factors from the adaptive immune pathway. Indeed, HMOs exposure induce a shift of the balance Th1/Th2 towards a Th1 polarization. Effects of some HMOs on T cell and DC cytokine secretion were also described and one study demonstrated that 2’-FL *in vivo*, increase regulatory T cells. Effects of HMOs on other major actors of the adaptive immune response need to be more investigated such has CD8 T cells and B cells. The modulation of the regulatory B cells were not investigated yet. Finally, studies are only observational and the molecular mechanism of HMOs leading to the regulation of the adaptive immune pathway (cell rate, cytokine secretion, cells interaction) still have to be deciphered.

Thus, because HMOs participate in the establishment of controlled inflammatory innate immunity and adaptative immunity and a tolerogenic environment, they could protect children from infections and allergic and autoimmune diseases.

## HMOs as a New Potential Therapeutic Agent

Because HMOs present many beneficial effects proven *in vitro* and *in vivo*, several studies have been conducted to confirm these beneficial effects against infections and inflammatory diseases. Therapeutic strategies used in clinical trials targeted different windows of opportunity of HMO supplementation to mediate the beneficial effects, such as the early life period, especially for babies in terms of milk formula.

### HMOs Tolerance and Health Effect

It was first necessary to confirm that HMOs were well tolerated and had no side effects (118) (**Table 1**).

Several studies conducted on newborns fed HMO-supplemented formulas demonstrated digestive tolerance of HMOs and identical child growth (weight, length, and head circumference) compared to breastfed infants (12–14, 117) (**Table 1**). HMOs induce better sleep and a less important pathogens colonization leading to less colic and more *Bifidobacterium* present in the colon (14). Sprenger et al. (9) demonstrated that children breastfed for 4 months by secreting mothers (with a secretion of 2’-FL) had no difference in weight, size, or body mass index compared to breastfed children from nonsecretor mothers (**Table 1**). No difference in cranial box circumference was described (119). Finally, it was also shown in pigs that supplementation with 2’-FL improved cognitive performance and influenced brain development (120). All these studies highlight that HMOs do not induce side effects on newborns breastfed by secretor mother or fed with a HMOs-supplemented formula. Nevertheless, it would be interesting to follow these children up to a certain age to see if differences appear in the long term or not.

### HMOs Effects on Babies’ Ailments, Infections and Non-Communicable Diseases

Because HMOs do not present side effects, they were tested to determine whether they induce a reduction in babies’ ailments. Supplemented formula-fed infants who were cesarean born had softer stools and a lower incidence of colic at 4 months of life than nonsupplemented children (14) (**Table 1**). They had a shorter period of bronchitis at 4, 6, and 12 months, fewer respiratory tract infections and a reduction in antibiotic and antipyretic uses compared to nonsupplemented children (14). It would have been interesting in this study to compare the results obtained in the supplemented formula-fed group with babies fed by secretor mothers to compare the effects of natural HMOs intake versus supplemented formula intake. HMOs are also associated with a reduction in diarrhea induced by *Campylobacter* during the first 2 years of life of children fed breast milk with a high concentration of 2’-FL compared to those fed with a low level of 2’-FL in the milk highlighting also the importance of the concentration of HMOs in the breast milk (11) (**Table 1**). In parallel, an important study revealed a decrease in mortality in noninfected children born to HIV-infected mothers due to HMOs in breast milk compared to noninfected children who were not breastfed (11).

Several studies highlighted the protective effects of HMOs against infections. HMOs act as soluble receptors of bacteria or modulate target cells, inhibiting the pathogen-host cell interaction. Indeed, Angeloni et al. demonstrated that 3’SL exposure induces a change in the glycocalyx composition of Caco-2 cells (37). The expression of α2-3- and α2-6-linked sialic acid residues was downregulated on the cell surface, leading to a decrease in pathogen receptors on the host cell surface and thus a decrease in bacterial binding. A decrease in virus binding to DLD1 intestinal cells by HMOs was also highlighted (121). Interestingly, this particular effect of HMOs is not limited to intestinal cells. Indeed, Andersson et al. demonstrated that human milk inhibits the attachment of bacteria and viruses on pharyngeal and buccal epithelial cells (122). It was demonstrated that the anti-adhesive effect was due to the low molecular weight fraction of human milk, particularly to the oligosaccharides present in this fraction.

Furthermore, HMOs act as antimicrobial agents due to their ability to bind pathogens such as *Streptococcus* strains directly in the gut (123). In an *in vitro* study conducted by Weichert et al., the use of X-ray crystallography demonstrated that 2’-FL and 3’-FL are able to bind norovirus, linking in the HGBA pocket of the virus, and inhibiting its cytotoxic activity (124). They also demonstrated that HMOs can be direct substrates for pathogens, preventing infection of host cells. Finally, Lin et al. highlighted that sialic acid HMOs protect epithelial cells by preventing the internalization of *Escherichia coli* in UPEC cells *in vitro*, inhibiting the infection of the cells (125).

Finally, the anti-adhesive properties of HMOs also applied to some parasitic protozoa. For example, *Entamoeba histolytica*, (a protozoa that destroys the epithelium of the large intestine and can occur in the liver, lungs or spleen) requires for the infection to attach to the host’s colon mucosa. Parasites that cannot attach are excreted in faeces and do not cause disease. It was shown that some HMOs significantly reduce the binding and cytotoxicity of *E. histolytica* during *in vivo* assays (118). This may explain why breastfed infants are less likely to be infected with *E. histolytica* or other protozoa than formula-fed infants. To summarize, HMOs can prevent infection by 1) modulating virus receptor expression on the surface of the cell, 2) directly binding pathogens inhibiting their adhesion, and 3) preventing internalization through the gut.

Finally, it was shown in animal models that HMOs had protective effects on several non-communicable diseases. 3’SL supplementation is able to prevent the development of obesity in mice and to prevent type 2 diabetes and cardiovascular diseases (126). In the same way, HMOs in breastmilk seem to protect children against obesity until 4 years of age compared to children who were never breastfed (119).

### HMOs Are a Potential Candidate to Reduce Allergic Diseases

Allergies are a major health issue in developed countries, and there is currently no effective therapeutic strategy to cope with allergies. Allergy pathologies are associated with a breakdown of immune tolerance (127), microbiota dysbiosis (128) and an increase in epithelial permeability (129). They may be observed within the first month of life, suggesting the possibility of early childhood preprogramming these biological systems and possibly reducing the risk of allergy development. Due to their beneficial effects on the microbiota, gut epithelial barrier and immune system, HMOs have been tested to treat or prevent allergic diseases. Today, only some preclinical and clinical studies have been conducted in this context.

### Preclinical Studies Using HMOs as a Therapeutic Strategy for Allergies

In a mouse model of food allergies, HMOs were shown to be good candidates to treat or prevent allergic symptoms. Indeed, allergic mice supplemented with 2’-FL had a decrease in allergic symptoms such as diarrhea and hypothermia and a suppression of protease 1 secretion from mast cells mediated by antigen compared to non-supplemented allergic mice (130). The authors also demonstrated a decrease in intestinal mast cell frequency and an increase in CD4^+^CD25^+^IL10^+^ regulatory T cells in mesenteric lymph nodes and Peyer’s patches. Furthermore, they observed a reduction in the passive response to skin anaphylaxis. Recently, Liu et al., working on a β-lactoglobulin-induced milk allergic mouse model, demonstrated that supplementation with 2’-FL for 4 weeks decreased IgE and β-lactoglobulin-specific IgE in the serum and increased the levels of the anti-inflammatory cytokines IL-10, TGF-β, and IFNγ compared to allergic mice untreated with 2’-FL, thus preventing allergy development (131).

These encouraging data from preclinical models need to be confirmed by other studies in other allergic contexts, such as asthma or atopic dermatitis, and the mechanistic effects of HMOs must be deciphered.

### Clinical Studies Evaluate the Interest of HMOs for Allergies

Most human studies have correlated the presence of HMOs in breastmilk and the development of allergies in infants, but the results are controversial.

On the one hand, Seppo et al. demonstrated that a high level of LNFPIII in breastmilk protected infants against cow’s milk allergy compared to children fed breastmilk containing a low level of LNFPIII (16). On the other hand, Miliku et al. showed that HMO composition in human milk is associated with food sensitization in the first year of life, which can later lead to food allergies (18). Lodge et al. demonstrated that compared with children exposed to the neutral Lewis HMO profile, exposure to acidic Lewis HMOs was associated with a higher risk of allergic disease and asthma during childhood, whereas exposure to the acidic-predominant profile was associated with a reduced risk of food sensitization (20). Finally, the observational clinical trial conducted by Sprenger et al. (132) revealed that infants born by cesarean section and having a high hereditary risk for allergies might have a lower risk of manifesting IgE-associated eczema at 2 years, but not at 5 years of age, when fed breast milk with FUT2-dependent milk oligosaccharides. Further studies with larger cohorts and especially randomized controlled intervention trials are required to build on these preliminary observations.

In an interventional study, Nowak-Wegrzyn et al. attempted to determine whether an extensively hydrolyzed formula supplemented with two HMOs was tolerated by infants allergic to cow’s milk (19). They demonstrated that the whey-based extensively hydrolyzed formula supplemented with 2’-FL and LNnT met the clinical hypoallergenic criteria and could be recommended for the management of cow’s milk protein allergy in infants and young children.

These different results obtained in clinical trials are contradictory because the studies were conducted in different ways (time of supplementation, concentration, type of HMOs), complicating the understanding of HMO effects on allergic disease. New strategies could be explored to clarify the HMO effect.

## Conclusion

HMO composition (presence/absence of neutral, acidic, and sialylated HMOs) and concentration in breastmilk depend on several factors, such as their different ramification and the stage of lactation. Another important factor is the mother secretor status. If she is Lewis (+) and secretor (+), all HMOs will be secreted, in particular 2’-FL and LNFPIII, which have protective effects on infant and adult health. 2’-FL and LNFPIII modulate the intestinal and fecal microbiota to establish beneficial bacterial colonization, limiting pathogenic bacterial infection and diseases. They are also able to directly bind to pathogens in the gut and to influence intestinal epithelial cells, inducing protection and reinforcement of the epithelial barrier. HMOs directly and indirectly activate the immune system, first activating an inflammatory reaction to prime the organism against infection and, second, activating a tolerogenic environment. Interestingly, HMOs molecular mechanism on the immune system, the epithelial barrier and gut microbiota seems to be very similar and can be compared to exogenous prebiotics such as galacto−oligosaccharides or inulin (133).

Due to the benefits of HMOs, they have been tested in several studies as potential treatments for different diseases, such as obesity, diabetes, virus infection (influenza, NEC) and allergies. Because the implementation of major systems occurs very early in life, even *in utero*, it would also be interesting to consider preventive strategies, by directly supplementing gestational mothers in HMOs. For example, maternal intake of omega-3, polyunsaturated fatty acid, antioxidants, folates, vitamin D or probiotics is associated with protection against allergic outcomes in children (134). In the context of prebiotics, two clinical studies are currently being conducted: SYMBA (Trial Identification Number: ACTRN12615001075572) and PREGRALL (Trial Identification Number: NCT03183440). SYMBA investigates the effects of maternal prebiotic supplementation from 18–20 weeks gestation during pregnancy until 6 months of lactation on the development of infant allergic disease (135). PREGRALL aims to evaluate the effectiveness of gestational prebiotic supplementation (from 20th week of gestation until delivery) on the occurrence of AD at 1 year of age in at-risk children (136). Because lactation is also a crucial period for the implementation of major systems, HMO supplementation of breastfed babies from non-secretor mother could be considered as preventive strategy for non-communicable diseases.

Despite a large number of publications showing the beneficial effects of HMOs on the host (such as protection against allergies), some results are quite controversial, especially due to the lack of homogeneity between all these studies in terms of the time of supplementation, concentration and type of HMOs. More experiments need to be performed to affirm the beneficial effect of HMOs on health.

## Author Contributions

AR and CB wrote the paper. SLG, HP, SB and MB reviewed the paper. All authors contributed to the article and approved the submitted version.

## Conflict of Interest

The authors declare that the research was conducted in the absence of any commercial or financial relationships that could be construed as a potential conflict of interest.
